# Cytotoxic Indole Alkaloids against Human Leukemia Cell Lines from the Toxic Plant *Peganum harmala*

**DOI:** 10.3390/toxins7114507

**Published:** 2015-11-03

**Authors:** Chunhua Wang, Zhenxue Zhang, Yihai Wang, Xiangjiu He

**Affiliations:** 1 School of Pharmacy, Guangdong Pharmaceutical University, Guangzhou 510006, China; E-Mails: wangchunhua@whu.edu.cn (C.W.); wangyihai@whu.edu.cn (Y.W.); 2 College of Chemical Engineering, Shenyang University of Chemical Technology, Shenyang 110142, China; E-Mail: zhangzhenxue02@hotmail.com

**Keywords:** *Peganum harmala*, indole alkaloids, cytotoxicity, anti-leukemia, mitochondrial pathway

## Abstract

Bioactivity-guided fractionation was used to determine the cytotoxic alkaloids from the toxic plant *Peganum harmala*. Two novel indole alkaloids, together with ten known ones, were isolated and identified. The novel alkaloids were elucidated to be 2-(indol-3-yl)ethyl-α-l-rhamnopyranosyl-(1 → 6)-β-d-glucopyranoside (**2**) and 3-hydroxy-3-(*N*-acetyl-2-aminoethyl)-6-methoxyindol-2-one (**3**). The cytotoxicity against human leukemia cells was assayed for the alkaloids and some of them showed potent activity. Harmalacidine (compound **8**, **HMC**) exhibited the highest cytotoxicity against U-937 cells with IC_50_ value of 3.1 ± 0.2 μmol/L. The cytotoxic mechanism of HMC was targeting the mitochondrial and protein tyrosine kinase signaling pathways (PTKs-Ras/Raf/ERK). The results strongly demonstrated that the alkaloids from *Peganum harmala* could be a promising candidate for the therapy of leukemia.

## 1. Introduction

Harmal (*Peganum harmala* L., Zygophyllaceae family) is a perennial, glabrous plant, which is widely distributed in Central Asia, North Africa and the Middle East. In China, it mainly grows in the northwest, such as Xinjiang and Inner Mongolia [1]. The whole plant of *Peganum harmala* (*P. harmala* L.) is a traditional medicine that has a long history of being widely used to treat apoplexia, asthma [2], jaundice and lumbago [3]. Recently, much research has revealed that β-carboline and quinazoline alkaloids are important ingredients, accounting for many of the pharmacological and therapeutic effects, such as analgesic [4], antibacterial [5], antiparasitic [6], strong reversible inhibition activity of monoamine oxidase [7], cytotoxic and antitumor activities, *etc.* [8,9].

In addition to the therapeutic effects, harmal also has some toxicity. There were several reports of human and animal intoxications induced by the plant [10,11]. There have been some toxic symptoms reported in different human cases following ingestions of its seed extract or infusion, such as neuro-sensorial symptoms, visual hallucinations, cardiovascular disorders such as bradycardia and low blood pressure, psychomotor agitation, diffuse tremors, ataxia and vomiting [12,13]. The extract of *P. harmala* could cause paralysis, liver degeneration, spongiform changes in the central nervous system, euphoria, convulsions, bradycardia, *etc.* [14,15].

A phytochemical work on the alkaloids from *P. harmala* L. was conducted to obtain two new and ten known compounds. The structures were elucidated by extensive spectroscopic techniques including IR, HR-ESI-MS, 1D and 2D NMR and specific rotation, as well as by comparison of the data with those in the literature. All alkaloids were evaluated for cytotoxicity against human leukemia cell lines (U-937, HL-60, KG1, and HEL). Moreover, cytotoxic mechanism of the alkaloids against human leukemia cells was investigated, and found that the alkaloids could induce apoptosis of leukemia cells by targeting the mitochondrial and protein tyrosine kinase (PTKs) signaling pathways.

## 2. Results

### 2.1. Structure Identification of the Purified Alkaloids

**Figure 1:**
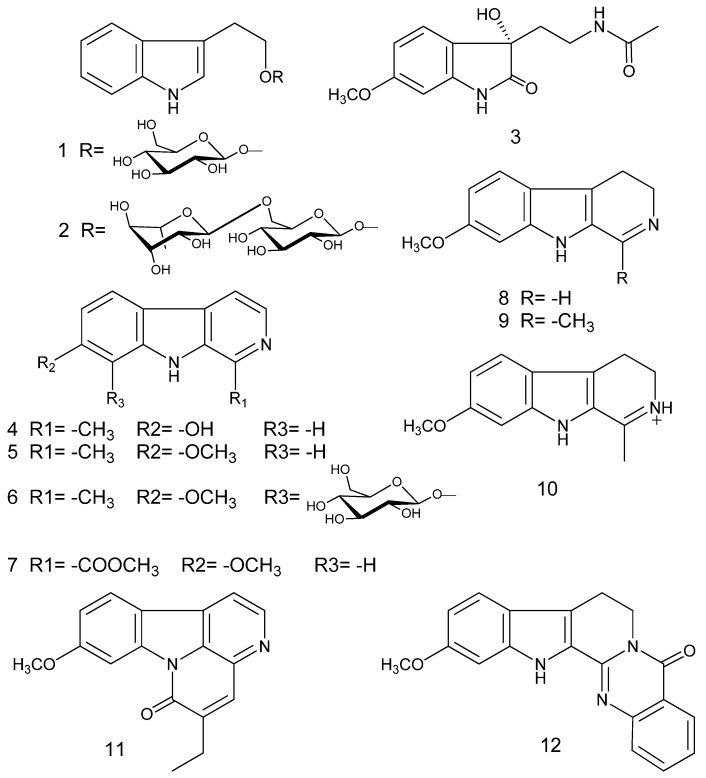
Alkaloids isolated from *P. harmala*.

The chemical structures of compounds **1**–**12** are shown in Figure 1. They were classified as indole alkaloids.

Compound **2** was isolated as a white amorphous powder. Its molecular formula was deduced as C_22_H_31_NO_10_ by HR-ESI-MS with the ion of *m*/*z* 470.2032 [M + H]^+^ (calcd. 470.2026). The ^1^H NMR spectrum indicated the present of four aromatic protons at δ_H_ 7.52 (1H, m), 7.22 (1H, m), 7.06 (1H, m), 6.97 (1H, m), which revealed a typical pattern for AAʹBBʹ splitting system of an ortho-disubstituted benzene ring. The anomeric protons at δ_H_ 4.23 (1H, d, *J* = 7.8 Hz) and 4.61 (1H, d, *J* = 1.0 Hz) suggested the presence of two sugar units in the molecule. There were eight sp^2^ hybrid carbon signals in the ^13^C NMR, which suggested the presence of an indole unit from the NMR data.

The NMR data of **2** were highly similar to those of compound **1**, which was identified as a known alkaloid of 2-(indol-3-yl)ethyl-β-d-glucopyranoside according to its spectroscopic data [16], except for the presence of an additional sugar unit (δ_C_ 100.9, 75.4, 72.1, 70.5, 70.2, 17.9), and the C-6ʹ signal at δ_C_ 67.1 in compound **2** had +6.0 ppm downfield-shift. The sugars were identified as d-glucose and l-rhamnose by GC analysis of their chiral derivatives after acid hydrolysis using authentic sugars as reference. d-glucose and l-rhamnose were detected in the relative proportion of 1:1. Therefore, compound **2** was assumed to be a glycoside of compound **1**. The linkage was established by the analysis of the HMBC correlations between H-6ʹ (δ_H_ 3.83) and C-1″ (δ_C_ 100.9), H-1″ (δ_H_ 4.61) and C-6ʹ (δ_C_ 67.1). Thus, the saccharidic chain of **2** was determined to be α-l-rhamnopyranosyl-(1 → 6)-β-d-glucosyl group. Based on the above analysis, the structure of compound **2** was established as 2-(indol-3-yl)ethyl-α-l-rhamnopyranosyl-(1 → 6)-β-d-glucopyranoside, and its chemical structure was shown in Figure 1.

Compound **3** was obtained as a white amorphous powder. Its molecular formula C_13_H_16_N_2_O_4_ was established on the basis of its HR-ESI-MS at *m*/*z* 287.0942 [M + Na]^+^ (calcd. 287.1008). In its IR spectrum, the absorptions at 3394, 1726, and 1635 cm^−1^ indicated the presence of NH and two carbonyls. The ^1^H NMR spectrum exhibited three aromatic protons at δ_H_ 7.16 (1H, d, *J* = 8.2 Hz), 6.52 (1H, dd, *J* = 8.2, 2.3 Hz) and 6.37 (1H, d, *J* = 2.3 Hz), which indicated the presence of an ABX spin system. It also had one methoxyl at δ_H_ 3.73 (3H, s), two methylenes at δ_H_ 2.88 (2H, m) and 1.86 (2H, m), and one methyl at δ_H_ 1.71 (3H, s). The ^13^C NMR spectrum displayed 13 resonances, which were classified by HSQC experiment as six aromatic carbons (δ_C_ 160.2, 142.9, 124.9, 123.6, 106.4, 96.6), one sp^3^ quaternary carbon (δ_C_ 74.1), one methoxyl (δ_C_ 55.3), two methylenes (δ_C_ 37.3, 33.8), one methyl (δ_C_ 22.6) and two amide carbonyls (δ_C_ 179.5, 168.9).

The key HMBC correlations of 1–NH singlet (δ_H_ 10.24) with C-2 (δ_C_ 179.5), C-3 (δ_C_ 74.1), C-4α (δ_C_ 123.6), C-7α (δ_C_ 142.9), and OH singlet (δ_H_ 5.86) with C-2 (δ_C_ 179.5), C-3 (δ_C_ 74.1), and C-4α (δ_C_ 123.6), suggested the existence of 2-oxo-3-hydroxy indole unit. Additionally, the HSQC and HMBC data of **3** defined the moiety of –CH_2_CH_2_NH– fragment, which were attached to the indole unit at C-3, supported by the HMBC correlations of H-8 (δ_H_ 1.86) with C-2 (δ_C_ 179.5), C-3 (δ_C_ 74.1), and C-4α (δ_C_ 123.6), and H-9 (δ_H_ 2.88) with C-3 (δ_C_ 74.1). Based on the above evidence, as well as the HMBC correlation of 10-NH (δ_H_ 7.73) with C-9 (δ_C_ 33.8), the other amide carbonyl (δ_C_ 168.9) led to identification of the partial structural fragment of –CH_2_CH_2_NHCOCH_3_. Therefore, the planar structure of compound **3** was established. The configuration of compound **3** was established as *S*-form by the specific rotation value of –8.2° (c 0.2, MeOH) and the related literature [17,18]. Based on above analyses, compound **3** was identified as (*S*)-3-hydroxy-3-(*N*-acetyl-2-aminoethyl)-6-methoxyindol-2-one, which was, as far as we know, a novel alkaloid.

Ten known alkaloids were identified as 2-(indol-3-yl)ethyl-β-d-glucopyranoside (**1**) [16], harmol (**4**) [19], harmine (**5**) [20], ruin (**6**) [21], harmic acid methyl ester (**7**) [22,23], harmalacidine (**8**) [24,25], harmaline (**9**) [26], protonated harmaline (**10**) [27], luotonin C (**11**) [28], and 11-methyoxyl-rutaecarpine (**12)** [29], by comparison of their spectroscopic data with the corresponding literature.

### 2.2. Cytotoxicity of the Alkaloids on Human Leukemia Cells

The alkaloids were assayed for cytotoxicity against human leukemia cell lines by MTT method, and their cytotoxicities were evaluated in parallel using cisplatin as the positive control. Meanwhile, the cytotoxicity against human embryonic kidney cells (HEK-293) was evaluated for their toxic selectivity.

The cytotoxic results are summarized in Table 1. Most of the alkaloids showed potent cytotoxicity against the four leukemia cells, with IC_50_ values less than 100 μmol/L. Among the alkaloids, compound **8** (harmalacidine, HMC) showed the highest potent cytotoxicity against U-937 cells, with an IC_50_ value of 3.1 ± 0.2 μmol/L. Meanwhile, the toxicities of all alkaloids against HEK-293 were much less than those of leukemia cells. The IC_50_ values against HEK-293 of compounds **1**, **2**, **3**–**5**, **8**, **11** and **12** were above 200 μmol/L. The results suggest that the alkaloids have perfect selectivity for human leukemia cells.

### 2.3. HMC Induced U-937 Cells Apoptosis

**Figure 2:**
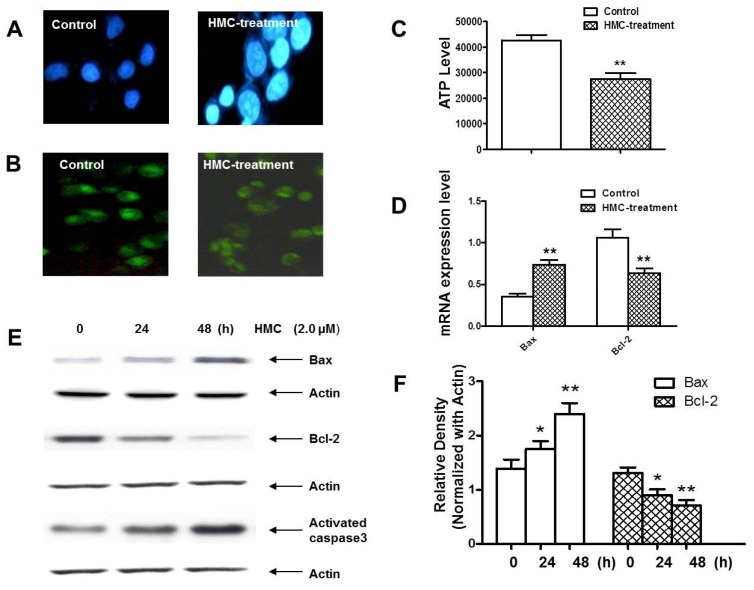
HMC initiated apoptosis in U-937 cells through mitochondrial and Caspase cascade. (**A**) Apoptotic U-937 cells were observed after the cells were stained by Hoechst 33258. (**B**) Mitochondrial transmembrane potential was reduced in U-937 cells stained by Rodamine123. (**C**) The ATP level was also reduced in U-937 cells. (**D**–**F**) HMC impacted the mRNA levels and protein expressions of Bax and Bcl-2 in U-937 cells leading to the activation of caspase 3. Data are presented as means ± SD of three independent tests. * *p* < 0.05 *versus* control, ** *p* < 0.01 *versus* control.

Based on the cytotoxic results, compound **8** (HMC) has pronounced cytotoxicity against human leukemia cells, especially to U-937 cells. Therefore, it was worthy of investigating the cytotoxic mechanism of HMC in U-937 cell death. To elucidate the characteristics of HMC-induced U-937 cells, morphologic changes were examined. Interestingly, when the U-937 cells were exposed to 2.0 μmol/L HMC for 24 h and stained by Hoechst 33258, the characteristic morphologic alterations of cell apoptosis were observed, including membrane blebbing, nuclear condensation and granular apoptotic bodies (Figure 2A).

**Table 1:** Cytotoxicity of the alkaloids from *P. Harmala* against human leukemia cells U-937, HL-60, KG1, and HEL, and human embryonic kidney cells HEK-293 (IC_50_, Mean ± SD, μmol/L).

Compound	IC_50_ (μmol/L)				
	U-937	HL-60	KG1	HEL	HEK-293
1	52.1 ± 2.6	78.9 ± 3.8	23.1 ± 1.4	121.3 ± 5.5	>200
2	80.2 ± 4.5	55.3 ± 3.4	60.2 ± 2.7	131.0 ± 4.9	>200
3	75.3 ± 2.8	36.2 ± 1.1	>200	55.3 ± 3.2	122.4 ± 5.7
4	46.1 ± 2.0	62.0 ± 2.8	47.7 ± 2.3	54.6 ± 2.3	>200
5	23.1 ± 0.9	71.2 ± 3.3	62.7 ± 2.8	20.3 ± 1.1	>200
6	30.6 ± 1.2	60.7 ± 2.9	44.5 ± 2.1	71.6 ± 2.7	>200
7	22.3 ± 1.4	44.3 ± 2.1	56.8 ± 2.5	35.2 ± 2.8	164.2 ± 8.1
8	3.1 ± 0.2	61.3 ± 2.8	32.6 ± 1.7	25.7 ± 1.1	>200
9	10.6 ± 0.7	55.3 ± 2.4	46.8 ± 1.9	20.1 ± 1.2	156.3 ± 7.2
10	15.3 ± 0.7	45.7 ± 2.1	56.9 ± 2.3	21.2 ± 1.1	132.5 ± 4.6
11	49.6 ± 1.9	87.3 ± 3.7	113.2 ± 5.6	68.3 ± 3.0	>200
12	55.3 ± 1.7	93.8± 6.8	88.1 ± 3.7	120.9 ± 5.5	>200
Cisplatin	6.3 ± 0.5	13.5 ± 0.4	4.1 ± 0.3	7.7 ± 0.2	120.6 ± 5.7

To make it clear whether HMC-induce apoptosis was related to mitochondrial function, mitochondrial transmembrane potential was assessed using Rhodamine 123. Interestingly, the result clearly showed that mitochondrial transmembrane potential was obviously reduced after HMC treatment compared with the control group (Figure 2B). Meanwhile, the ATP level of mitochondria was measured in U-937 cells treated by HMC. The result suggested that HMC might reduce the ATP level after 2.0 μmol/L HMC treatment for 24 h, accompanied by the reduction of mitochondrial transmembrane potential.

Since Bcl-2 family members were critical regulators of the mitochondrial pathway that induced intrinsic caspase activation, RT-PCR was applied to detect the mRNA level of Bax and Bcl-2 in HMC-treated U-937 cells. The results showed that HMC could increase the Bax mRNA and reduce Bcl-2 mRNA. Meanwhile, Western blot analyses were carried out to observe the expressions of Bcl-2 and Bax. After treated with HMC for 24 h, the expression of pro-apoptotic Bax began to increase, while anti-apoptotic Bcl-2 began to decrease accordingly. Meanwhile, the downstream Caspase 3 was activated, which was positively related with down-regulated Bax (Figure 2E,F).

### 2.4. HMC Induced U-937 Cells Apoptosis via Ras/Raf/ERK Pathway

**Figure 3:**
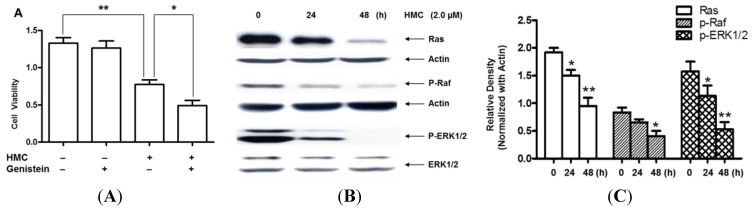
Apoptosis via Ras/Raf/ERK signaling pathway in U-937 cells after HMC treatment. (**A**) Cell viability was assessed using MTT method in U-937 cells after pretreated by 10 µmol/L Genistein for 1 h, and then the cells were incubated without or with 2.0 µmol/L HMC for 48 h. (**B**,**C**) Expressions of Ras, p-Raf and P-ERK1/2 were detected using Western blot analysis and the quantity results were showed in Figure 3C. Data are presented as means ± SEM of three independent tests. * *p* < 0.05 *versus* control, ** *p* < 0.01 *versus* control.

Unusual activation of Ras/Raf/MAPK signaling pathway is one of main characteristic of human cancer. In this study, the Ras/Raf/MAPK pathway was investigated in HMC-induced U-937 cell apoptosis. Genistein, a broad-spectrum inhibitor of protein tyrosine kinases (PTKs), was used to detect the cell viability after HMC treatment. As expected, genistein could significantly decrease the cell viability after HMC co-incubation compared with HMC-treated only (Figure 3A). Moreover, the expressions of Ras/Raf/ERK in U-937 cells were detected after HMC treatment in different time courses. The results strongly suggested that HMC could down-regulate the expressions of Ras, p-Raf and p-ERK1/2 (Figure 3B,C), while the total ERK kept no change.

## 3. Discussion

In this study, 12 indole alkaloids were purified and identified from *P. harmala*. 2-(indol-3-yl)ethyl-α-l-rhamnopyranosyl-(1 → 6)-β-d-glucopyranoside and 3-hydroxy-3-(*N*-acetyl-2-aminoethyl)-6-methoxyindol-2-one are, as far as we know, two novel alkaloids. Most alkaloids exhibited obvious cytotoxicity against leukemia cells. Meanwhile, the antitumor mechanism of HMC was investigated owning to its high selection in U-937 cells.

The study showed that HMC could inhibit the proliferation of U-937 cells, which was involved in apotosis. In HMC-induced apoptosis, the membrane potential and ATP level of mitochondria decreased dramatically in leukemia cells. HMC could up-regulate the expression level of Bax mRNA, while the Bcl-2 mRNA was down-regulated. The related protein expressions matched with the mRNA level pretty well. These results suggested that changes in the ratio of pro-apoptotic and anti-apoptotic Bcl-2 family proteins might contribute to the apoptosis promotion activity of HMC.

The small G-protein Ras, or its downstream effector Raf, could link receptor and non-receptor tyrosine kinases to downstream serine/threonine kinases. Ras activates a number of pathways. Ras/Raf/MEK/ERK pathway has different effects on growth, prevention of apoptosis, cell cycle arrest and induction of drug resistance in cells through various lineages. In this study, HMC could reduce the cell viability after pretreatment with genistein. The expressions of Ras, p-Raf, P-ERK1/2 were also reduced in U-937 cells. These results suggested that HMC inactivated Ras/Raf/ERK pathway to inhibit cell proliferation.

## 4. Experimental Section

### 4.1. Plant Materials

The seeds of *P. harmala* were collected in Kalpin County, Xinjiang, China, in 2012, and identified by Prof. Xiangjiu He, the School of Pharmacy, Guangdong Pharmaceutical University. The specimen (No. GDPU-NPR-2013001) was deposited in the Department of Medicinal Chemistry, Guangdong Pharmaceutical University.

### 4.2. Reagents and Chemicals

All chemicals used in the chemical study, such as acetone, dichloromethane, ethyl acetate, hexane, methanol and *n*-butanol, were of analytical grade and were purchased from Guangzhou Chemical Company (Guangzhou, China). The deuteriated dimethyl sulfoxide for NMR measurement was purchased from Sigma-Aldrich, Inc. (St. Louis, MO, USA).

Silica gel for column chromatography, 300–400 mesh, was purchased from Liangchen Silicon Material Co. (Lu’an, China). Precoated Rp-18 TLC plates were obtained from Macherey-Nagel (Düren, Germany). Octadecylsilane (ODS) for medium pressure liquid chromatography (MPLC) was purchased from Sigma-Aldrich Chemical Co. (St. Louis, MO, USA). The semi-preparative HPLC column was product of Cosmosil (5C18-MS-II, 20 ID × 250 mm, Nacalai, Nakagyo-ku, Japan).

Caspase-8, Caspase-3, Fas, FasL and Caspase-3 activity kit were purchased from Cell Signaling Technology (Danvers, MA, USA). Hoechst 33258 were purchased from Beyotime Institute of Biotechnology (Suzhou, China). Thiazolyl blue tetrazolium bromide (MTT) was purchased from Sigma-Aldrich Chemical Co. (St. Louis, MO, USA).

### 4.3. Instrumentation

The IR spectra were obtained on a PerkinElmer Spectrum 100 FT-IR spectrometer (Waltham, MA, USA). The NMR spectra were recorded on a Bruker AV-500 spectrometer (Bremen, Germany), using TMS as an internal standard. The ESI-MS were measured with a Waters ESI-Q-TOF-MS spectrometer (Milford, MA, USA). Semi-preparative HPLC was carried out on a Waters 600 system with a PDA detector (Milford, MA, USA).

### 4.4. Extraction, Fractionation and Purification

The dried seeds of *P. harmala* (11 kg) were powdered and extracted with 70% aqueous methanol three times. After evaporation of the solvent under reduced pressure, the residues (2.3 kg) were suspended in water and acidified to pH 1.0 with 10% hydrochloric acid. Lipophilic impurities were removed with CHCl_3_ extraction and the aqueous fraction was alkalized to pH 7.0 with NH_3_·H_2_O. The total alkaloids were extracted four times with CHCl_3_ and *n*-BuOH sequentially, and produced chloroform fraction (220 g) and butanol fraction (280 g), respectively.

The chloroform fraction (200 g) was subjected to a silica gel chromatography (300–400 mesh, 1650 × 100 mm) eluted with a CH_2_Cl_2_/MeOH gradient elution (containing 0.1% triethylamine). Compounds **5** (18.0 g) and **9** (15.0 g) were recrystallized and purified from CH_2_Cl_2_/MeOH 50:1 and 20:1 elution. Compounds **8** (15.0 mg), **3** (20.0 mg) and **7** (10.0 mg) were obtained from CH_2_Cl_2_/MeOH 100:1, 40:1 and 20:1 fractions after repeated silica gel and Sephadex LH-20 columns, respectively.

The butanol fraction (250 g) was separated into 13 fractions (B1–B13) by silica gel column (300–400 mesh, 100 × 1500 mm) eluted with CHCl_3_/MeOH/Triethylamine (100:0:0.1 to 0:100:0.1, *v*/*v*/*v*), in increasing order of polarity. Fraction B1 (2.1 g) was subjected to silica gel chromatography and eluted with cyclohexane/ethyl acetate (100:0 to 50:50) successively to yield compound **12** (5.0 mg). Fraction B2 (2.2 g) was purified by silica gel column, followed by Sephadex LH-20, and produced compound **11** (20.0 mg). Compound **10** (3.8 g) was recrystallized from fraction B6 (40.0 g) using methanol. Fraction B10 (7.2 g) was subjected to ODS MPLC, followed by a semi-preparative HPLC using the Cosmosil packed column (5C18-MS-II, 20 ID × 250 mm), and obtained compounds **1** (22.0 mg) and **4** (15.0 mg). Compounds **2** (30.0 mg) and **6** (20.0 mg) were purified from the fractions B11 (3.7 g) and B12 (10.6 g) through ODS MPLC and prepared HPLC.

2-(Indol-3-yl)ethyl*-*α*-*l*-*rhamnopyranosyl-(1 → 6)-β-d-glucopyranoside (**2**), white amorphous powder, positive to Dragendroff reagent. ^1^H NMR (500 MHz, DMSO-*d*_6_): δ_H_ 10.78 (s, –NH), 7.51 (d, *J* = 7.8 Hz, H-4), 7.33 (d, *J* = 8.1 Hz, H-7), 7.22 (d, *J* = 2.1 Hz, H-2), 7.06 (m, H-6), 6.98 (m, H-5), 4.74 (d, *J* = 7.8 Hz, Glc-1′), 4.61 (d, *J* = 1.0 Hz, Rha-1″), 3.97 (m, H-9), 3.83 (m, Glc-6′), 3.72 (m, H-9′), 3.63 (m, Glc-4′), 3.47 (m, Rha-5″), 3.44 (m, Rha-2″, Rha-3″), 3.30 (m, Glc-5′), 3.19 (m, Rha-4′′), 3.17 (m, Glc-3′), 3.16 (m, H-8), 2.97 (m, H-8′), 2.99 (m, Glc-2′), 1.13 (d, *J* = 6.2 Hz, Rha-6″). ^13^C NMR (125 MHz, DMSO-*d*_6_): δ_c_ 136.1 (C-7a), 127.3 (C-3a), 123.1 (C-2), 120.9 (C-6), 118.4 (C-5), 118.3 (C-4), 111.4 (C-7), 110.9 (C-3), 103.0 (Glc-1′), 100.9 (Rha-1″), 76.9 (Glc-3′), 75.4 (Glc-5′), 73.5 (Glc-2′), 72.4 (Rha-4″), 70.7 (Rha-3″), 70.5 (Rha-2″), 70.2 (Glc-4′), 69.4 (C-9), 68.4 (Rha-5″), 67.1 (Glc-6′), 25.5 (C-8), 17.9 (Rha-6′′).

(*S*)-3-Hydroxy-3-(*N*-acetyl-2-aminoethyl)-6-methoxyindol-2-one (**3**), white amorphous powder, positive to Dragendroff reagent. ^1^H NMR (500 MHz, DMSO-*d*_6_): δ_H_ 10.24 (s, –NH), 7.16 (d, *J* = 8.2 Hz, H-4a), 6.52 (dd, *J* = 8.2, 2.3 Hz, H-5), 6.37 (d, *J* = 2.3 Hz, H-7), 3.73 (s, –OCH_3_), 2.88 (m, H-9), 1.86 (m, H-8), 1.71.(s, CH_3_CO=). ^13^C NMR (125 MHz, DMSO-*d*_6_): δ_c_ 179.5 (C-2), 168.9 (CH_3_CO=), 160.2 (C-6), 142.9 (C-7a), 124.9 (C-4), 123.6 (C-4a), 106.4 (C-5), 96.6 (C-7), 74.1 (C-3), 55.3 (–OCH_3_), 37.3 (C-8), 33.8 (C-9), 22.6 (CH_3_CO=).

### 4.5. Cell Lines and Cultures

Human leukemia cell lines (U-937, HL-60, KG1 and HEL) and human embryonic kidney cells (HEK-293) were purchased from American Type Culture Collection (ATCC) (Manassas, VA, USA). Cells were cultured in RPMI 1640 supplemented with 10% fetal bovine serum (Gibco Life Technologies, Grand Island, NY, USA), 50 U/mL penicillin and 100 μg/mL streptomycin and maintained in a humidified atmosphere containing 5% CO_2_ at 37 °C. Penicillin and streptomycin were purchased from Hyclone (Logan, UT, USA).

### 4.6. Cytotoxicity Assay

Cytotoxicity was measured by the MTT assay. Briefly, cells were cultured in RPMI 1640 supplemented with 10% fetal bovine serum, 10 mmol/L Hepes, 50 units/mL penicillin, 50 μg/mL streptomycin, and 100 μg /mL gentamicin and were maintained at 37 °C in 5% CO_2_. A total of 2.5 × 10^4^ cells in growth media were placed in each well of a 96-well flat-bottom plate. After 12 h of incubation, the growth medium was replaced by media containing different concentrations of the test samples. Cytotoxicity was determined at 48 h from the MTT absorbance (490 nm) reading for each concentration compared to the control. At least three replications for each sample were used to determine the cytotoxicity.

### 4.7. Detection ATP Level in U-937 Cells

ATP luminescence was detected according to the manufactured information to evaluate the mitochondrial damage after 2.0 µmol/L HMC insulting. U-937 cells were treated without or with HMC for 24 h. After incubation, equilibrate the plates and its contents at room temperature for 30 min. One hundred microliters of cell titer reagent was added to each well in a 96-well plate and the plate was shaken gently for 2 min to induce cell lysis. The plate was incubated at room temperature for 10 min to stabilize luminescent signal and then record luminescence.

### 4.8. Real-Time PCR Analysis

Total RNA extraction for leukemia cells was performed by using Trizol method. U-937 cells were seeded in 6 well plates for 24 h, and then the cells were treated by 2.0 µmol/L HMC for another 24 h. After treatment, the cells were collected for RNA extraction. The forward and reversed primers of Bax were CAAGAAGCTGAGCGAGTGTCT and CAATCATCCTCTGCAGCTCCATATT. The forward and reversed primers of Bcl-2 were TGCGCTCAGCCCTGTG and GGTAGCGACGAGAGAAGTCATC. The forward and reversed primers of GAPDH were ACCCACTCCTCCACCTTTG and CTCTTGTGCTCTTGCTGGG. The reaction was conducted under the conditions with 94 °C for 30 s, 95 °C for 5 s, 62 °C for 30 s, 72 °C for 30 s for 30 cycles. Identity of the PCR-products was controlled by melting curve analysis. Standard curves were prepared from isolated PCR products by serial dilution. Data were normalized towards GAPDH expression and statistically analyzed.

### 4.9. Western Blot Analysis

U-937 cells were treated with 2.0 µmol/L HMC for 0, 24 and 48 h, respectively. The whole cells were collected, and then the cell pellets were resuspended with RIPA buffer that contained a mixture of protease inhibitors at 4 °C for 30 min. After 14,000× *g* centrifugation for 15 min, the protein content of supernatant was determined by the Bio-Rad DC protein assay (Bio-Rad Laboratories, Hercules, CA, USA). Fifteen micrograms of the total proteins from each sample were separated by 12% SDS-PAGE and transferred to nitrocellulose membranes, the membranes were soaked in blocking buffer (5% skimmed milk). Proteins were detected using primary antibodies, followed by HRP-conjugated secondary antibody. Labeling was detected using the ECL system (Amersham Biosciences Corp., Piscataway, NJ, USA).

### 4.10. Statistical Analysis

Data were expressed as the mean ± SD. Analyses were performed using Prism software with a two-way ANOVA to identify significant effects. Differences were considered significant at *p* < 0.05, *n* = 3.

## 5. Conclusions

Twelve indole alkaloids, including two novels, were purified and identified from *P. harmala*. The chemical structures were determined by spectroscopic and chemical methods. The cytotoxicities against five human leukemia cell lines were assayed for the alkaloids. Some alkaloids showed potent cytotoxicity against human leukemia cells. Harmalacidine (HMC) showed the highest cytotoxicity against U-937, which could induce cell apoptosis. The transmembrane potential and ATP level of mitochondria were obviously reduced after HMC treatment. HMC could also increase the Bax mRNA, while the Bcl-2 mRNA was down-regulated. It could down-regulate the expressions of Ras, p-Raf and p-ERK1/2, while the total ERK kept no change. These results suggested that HMC inactivated Ras/Raf/ERK pathway to inhibit U-937 proliferation, and may be a novel candidate for the therapy of human leukemia.
